# Corrigendum to: Ebselen induces reactive oxygen species (ROS)‐mediated cytotoxicity in *Saccharomyces cerevisiae* with inhibition of glutamate dehydrogenase being a target https://doi.org/10.1016/j.fob.2014.01.002

**DOI:** 10.1002/2211-5463.13785

**Published:** 2024-03-01

The above article contains duplicated panels in Figs 4 and 7. In Fig. 4, the differential interference contrast (DIC) micrographs of gdh3 Δ /gdh3 Δ in panel A and wild‐type in panel B are identical. As the authors no longer have the original data, the DIC micrographs have been removed from the amended version of Fig. 4 below.

**Fig. 4:**
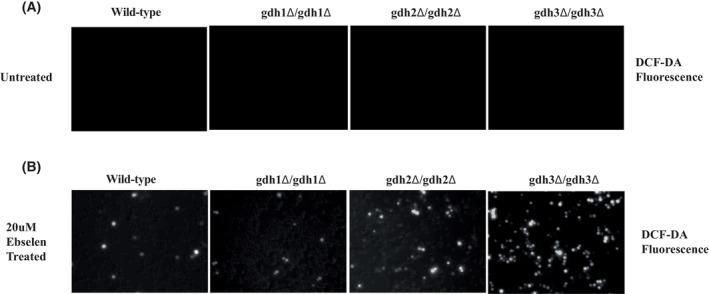
gdh3‐dependent reactive oxygen species (ROS) generation by ebselen in *S. cerevisiae*. Wild‐type (WT) and the gdh1‐, gdh2‐, and gdh3‐deletion mutant were treated with 20 μm ebselen for 2 h. Then, they were stained with 10 μm H2DCFDA for 1 min, and the level of ROS was observed by fluorescence microscopy. (A) Background ROS levels in WT, gdh1‐, gdh2‐, and gdh3‐deletion mutants. (B) ROS level in mutant and WT cells.

In Fig. 7C, the micrographs taken at 0 h for both the dimethyl sulfoxide (DMSO) control and the ebselen experiment are identical. The authors explain that this is because a single population of cells was divided into a control and experimental group after the 0 h micrograph was acquired. Figure 7 has been amended to indicate that the control and experimental groups were identical at the 0 h time point.

**Fig. 7:**
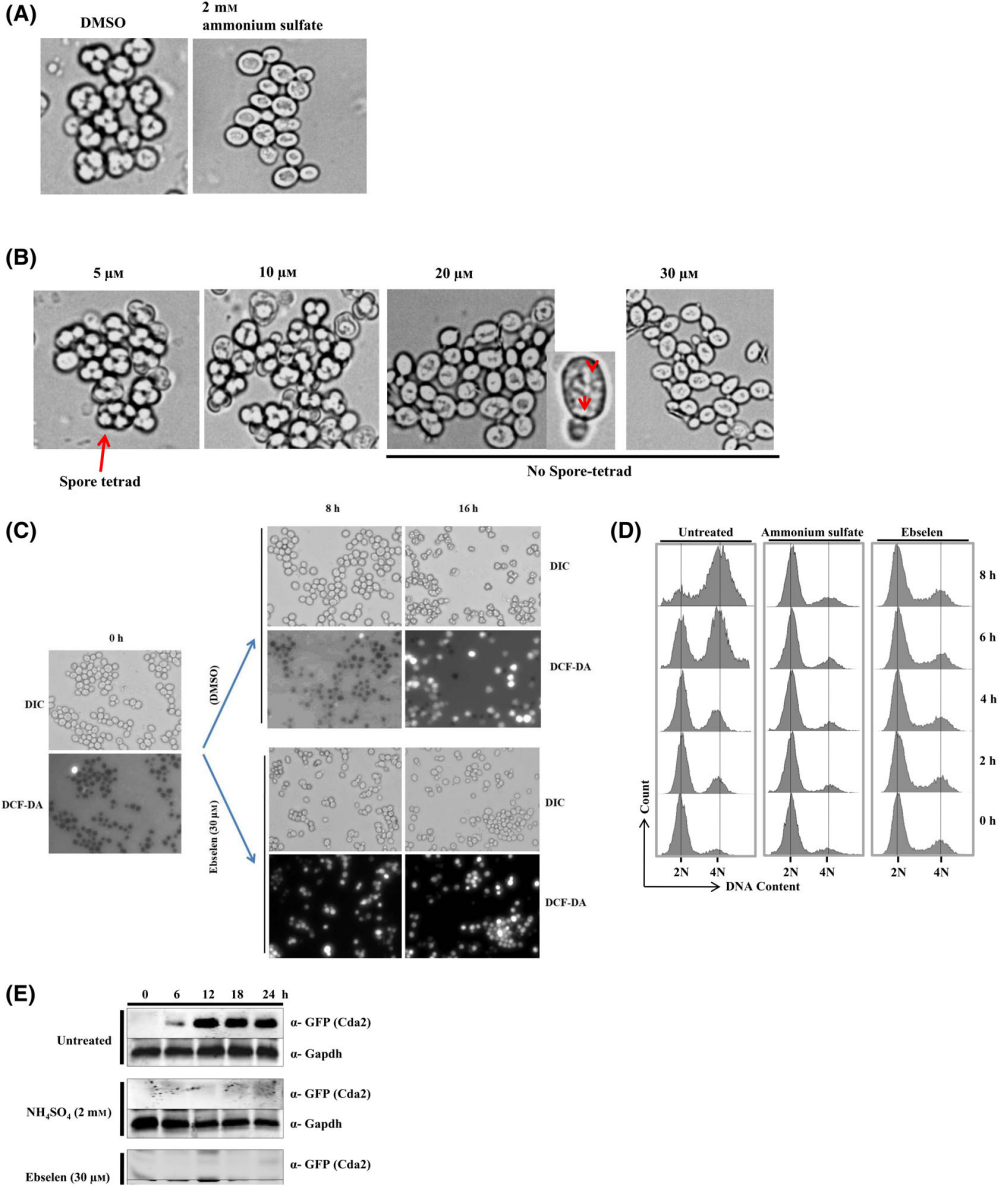
Ebselen strongly inhibits sporulation in yeast. (A) Microscopic images of cells sporulated for 24 h in the absence of drug (control), in the presence of 2 mm ammonium sulfate, (B) or increasing concentration of ebselen. Part of the 20 μm ebselen treated image was magnified; arrows indicate granular bodies of unknown origin. (C) Microscopic images of cells sporulated for the indicated time (0, 8, and 16 h) in the absence of drug (dimethyl sulfoxide (DMSO)), in the presence of 30 μm ebselen. The 0 h time point is the same for both the DMSO and ebselen treatment conditions. The upper panels show phase contrast microscopy; the lower panels show fluorescence microscopy of the same cells after staining with DCF‐DA. (D) Analysis of premeiotic DNA synthesis in a control (DMSO), and cells treated with ammonium sulfate (2 mm) or ebselen (30 μm) through FACS. Samples were taken at regular intervals as indicated in figure after induction of sporulation. Samples were subjected to FACS analysis and results were processed with BD FACS Diva software. (E) Yeast strain USY613 (USY61+ pCDA2‐eGFP::HygB) was cultured as described in materials and methods and treated with 30 μm of ebselen or 2 mm ammonium sulfate for 24 h. Ten‐milliliter cells were harvested at regular intervals (0, 6, 12, 18, and 24 h). Whole‐cell extracts were prepared by the TCA extraction method, and samples were subjected to Western blot analysis using indicated antibodies. Tbp and Gapdh served as loading controls.

These changes do not affect the conclusions of the paper.

